# GPAT3 is a potential therapeutic target to overcome sorafenib resistance in hepatocellular carcinoma

**DOI:** 10.7150/thno.92646

**Published:** 2024-06-01

**Authors:** Yu Zhou, Huakan Zhao, Ran Ren, Mingyue Zhou, Jiangang Zhang, Zhijuan Wu, Yu Chen, Juan Lei, Yang Chen, Ying Yu, Yongsheng Li

**Affiliations:** 1 Department of Medical Oncology, Chongqing University Cancer Hospital, Chongqing 400030, China.; 2 College of Pharmacy and Bioengineering, Chongqing University of Technology, Chongqing 400054, China.; 3 Chongqing University Cancer Hospital, School of Medicine, Chongqing University, Chongqing 400044, China.

**Keywords:** sorafenib resistance, triglyceride, GPAT3, hepatocellular carcinoma, apoptosis

## Abstract

**Background:** Sorafenib is the standard treatment for advanced hepatocellular carcinoma (HCC), but acquired resistance during the treatment greatly limits its clinical efficiency. Lipid metabolic disorder plays an important role in hepatocarcinogenesis. However, whether and how lipid metabolic reprogramming regulates sorafenib resistance of HCC cells remains vague.

**Methods:** Sorafenib resistant HCC cells were established by continuous induction. UHPLC-MS/MS, proteomics, and flow cytometry were used to assess the lipid metabolism. ChIP and western blot were used to reflect the interaction of signal transducer and activator of transcription 3 (STAT3) with glycerol-3-phosphate acyltransferase 3 (GPAT3). Gain- and loss-of function studies were applied to explore the mechanism driving sorafenib resistance of HCC. Flow cytometry and CCK8 in *vitro,* and tumor size in *vivo* were used to evaluate the sorafenib sensitivity of HCC cells.

**Results:** Our metabolome data revealed a significant enrichment of triglycerides in sorafenib-resistant HCC cells. Further analysis using proteomics and genomics techniques demonstrated a significant increase in the expression of GPAT3 in the sorafenib-resistant groups, which was found to be dependent on the activation of STAT3. The restoration of GPAT3 resensitized HCC cells to sorafenib, while overexpression of GPAT3 led to insensitivity to sorafenib. Mechanistically, GPAT3 upregulation increased triglyceride synthesis, which in turn stimulated the NF-κB/Bcl2 signaling pathway, resulting in apoptosis tolerance upon sorafenib treatment. Furthermore, our *in vitro* and *in vivo* studies revealed that pan-GPAT inhibitors effectively reversed sorafenib resistance in HCC cells.

**Conclusions:** Our data demonstrate that GPAT3 elevation in HCC cells reprograms triglyceride metabolism which contributes to acquired resistance to sorafenib, which suggests GPAT3 as a potential target for enhancing the sensitivity of HCC to sorafenib.

## Introduction

According to incomplete statistics from 2023, liver cancer remains a common cause of cancer-related deaths 1. Hepatocellular carcinoma (HCC) is the most prevalent primary liver cancer worldwide. Unfortunately, a majority of HCC patients are diagnosed at an advanced stage. While hepatectomy and liver transplantation are effective treatments, they are not suitable for advanced patients and have limitations based on the patient's condition 2. Sorafenib, a multi-kinase inhibitor, is a first-line targeted drug for treating advanced HCC 3, 4. However, only approximately 30% of advanced HCC patients benefit from sorafenib, and this population typically develops resistance to the drug within 6 months 5. Therefore, it is crucial to understand and study the mechanisms and effective prevention approaches for sorafenib resistance in order to improve the therapeutic outcomes for advanced HCC.

Lipids, specifically triglyceride (TAG), are vital nutrients that provide energy and materials for cells 6, 7. Lipid metabolism plays a significant role in various diseases, and increased lipid synthesis or uptake can accelerate the development of HCC 8, 9. Analysis of plasma lipids in patients before and after sorafenib treatment showed significant changes 10. Conversion of glycerol-3-phosphate and long-chain acyl-CoA to lysophosphatidic acid, the first step in *de novo* triglyceride synthesis, is catalyzed by glycerol-3-phosphate acyltransferases (GPATs). As the main GPAT subtype that affects TAG synthesis, GPAT3 has been reported that involved in regulating the proliferation, invasion, metastasis and chemotherapy-resistant in breast cancer 11, 12. Whereas the function of GPAT3 in HCC cells or sorafenib resistance remains unclear.

The decomposition of induced LD and mitochondrial lipid toxicity in HCC xenografts reduced cell resistance to sorafenib 13, however, the underlying mechanism by which lipid metabolic reprogramming driving sorafenib resistance of HCC is largely unknown. Here, we aimed to explore the molecular basis of lipid metabolic reprogramming in regulating sorafenib resistance in HCC cells.

## Materials and methods

### Information of HCC patients

The use of clinical information in this study was approved by the Ethics Committee of Chongqing University Cancer Hospital, and informed written consent was obtained from all participants. The clinicopathological characteristics of HCC patients were summarized in Table S1.

### Cell lines and cell culture

Hep3B (RRID: CVCL_0326), MHCC97H (RRID: CVCL_4972), and HEK-293T (RRID: CVCL_0063) cell lines were obtained from the GuanDao Biological Engineering Co., Ltd (Shanghai, China). The authenticity of the cell lines was verified, and they were tested for mycoplasma contamination. The cells were cultured in high glucose DMEM medium (Gibco, Grand Island, NY, USA) supplemented with 10% fetal bovine serum (Gibco) and 1% penicillin-streptomycin solution (Beyotime Biotechnology, Shanghai, China) at 37 °C in a humidified atmosphere with 5% CO_2_.

### Mice

Four-week-old male B-NDG® mice were purchased from Biocytogen (Beijing, China) and housed in a specific pathogen-free room. All animal studies were conducted in accordance with the relevant guidelines of the ethics committee of Chongqing University Cancer Hospital, following the National Institutes of Health Guidelines for animal welfare. To establish the tumor formation model, each subgroup of cells (5 × 10^6^) suspended in 100 μL of serum-free PBS were subcutaneously injected into the right armpit of mice in each group. In the experiments involving the administration of sorafenib to different cells, sorafenib was injected intraperitoneally at a dose of 2 mg/kg, once every 3 days, when the tumor volume reached 50 mm^3^. Tissue samples were collected after 7 injections. In the experiment using a subcutaneous tumor-bearing mouse model with MHCC97H SR cells under different treatments, sorafenib alone or in combination with pan-GPAT inhibitors was intraperitoneally injected at doses of 2 mg/kg and 1 mg/kg, respectively, once every 2 days, when the tumor volume reached 100 mm^3^. Tissue samples were collected after 7 injections.

### Proteomic assay analysis

The MHCC97H parent and SR cells (1 × 10^7^) were collected in cold PBS and transported on dry ice to Nanjing Aoyin Biotechnology Co., Ltd. (Nanjing, China) for proteomic analysis. The detection process involved protein extraction, protease digestion, iTRAQ labeling, and subsequent mass spectrometry analysis. Proteins expression was shown in Table S2-3.

### CRISPR/Cas9 targeted deletion of GPAT3 in HCC-SR cells

To knockout the *GPAT3* gene in MHCC97H-SR and Hep3B-SR cells, we utilized guided RNA (sgRNA) sequences (Cat. No. HCP260234-CG12-1; GeneCopoeia) specific to the human GPAT3 gene. Lentivirus was used to infect MHCC97H and Hep3B sorafenib resistant cells for 48 h, followed by selection with G418 (400 μg/mL) for a duration of 1 month to establish monoclonal cells. The GPAT3 deletion cells were subsequently analyzed using western blotting.

### GPAT3 over-expression in HCC cells

To over-express the GPAT3 gene in MHCC97H and Hep3B cells, we utilized a human *GPAT3* lentivirus (Cat. No. EX-Z1294-Lv152; GeneCopoeia). The lentivirus and control vector were generated in HEK-293T cells following the instructions provided in the Lenti-PacTM HIV Expression Packaging Kit (Cat. No. LT001; GeneCopoeia). After 72 h of transfection, the lentivirus was harvested and filtered through a 0.45 μm filter prior to infection. The HCC cells were infected with the virus three times, with each infection lasting for two days. Following infection, the cells were selected with Hygromycin (200 μg/mL) for one month, and subsequently analyzed using western blotting.

### GPAT3 knockdown in HCC cells

To knockdown the *GPAT3* gene in Hep3B cells, lentiviruses containing shRNA (NC sequence: TTCTCCGAACGTGTCACGT; 1^#^ sequence: CCCAAAGGAGTCGATTCTTAA; 2^#^ sequence: CCTGGTTACTAAGAGACTAAA) were used to infect Hep3B cells for 24 h, followed by selection with puromycin (4 μg/mL) for a duration of 1 month to establish monoclonal cells. The knockdown efficiency of *GPAT3* evaluated by RT-qPCR.

### Cell counting kit-8 (CCK-8) assay

The CCK-8 kit was purchased from Dojindo Laboratories (Dojindo, Kumamoto, Japan). A suspension of 3 × 10^4^ MHCC97H or 5 × 10^4^ Hep3B cells/mL was inoculated into a 96-well plate at a volume of 100 μL per well and incubated overnight at 37 °C, various treatments were administered after the cells were allowed to attach overnight. Cell cytotoxicity was assessed after 72 hours by adding CCK-8 solution. In the cell proliferation experiment, CCK-8 working solution was added every 24 hours to analyze the samples. The plate was then incubated at 37 °C for 2 h, as per the instructions. The absorbance was measured at 450 nm using the Varioskan Flash Spectral Scanning Multimode Reader (Thermo Fisher Scientific, Waltham, MA, USA).

### Real-time quantitative PCR (qPCR) assay

A total of 2 × 10^5^ cells were seeded into each well of a 6-well plate with the indicated treatments. Cell RNA was extracted using RNAiso Plus (Cat. No. 9109, TaKaRa, Otsu, Shiga, Japan) following the manufacturer's instructions. The RNA was then reverse transcribed into cDNA using the PrimeScript RT reagent Kit with gDNA Eraser (Cat. No. RR047A, TaKaRa). mRNA expression was assessed by qPCR using SYBRII (Cat. No. B21403, Bimake) on the CFX384 system (Bio-Rad, CA). Actin was used as the internal control for PCR amplification to measure the expression of target genes. The 2^-ΔΔCt^ method was employed to compare and quantify the target genes, and the analysis was performed using Excel. The primers used are listed in Table S4.

### Western-blotting assay

After washing the prepared cell samples twice with ice-cold PBS, the total cellular proteins were extracted. Each lane was then added with 40 μg protein samples and separated by 4%-12% Bis-Tris PAGE electrophoresis. The separated proteins were transferred to a 0.2 μm PVDF membrane for detection. Next, the membranes were blocked in tris-buffered saline containing 0.05% Tween 20 (TBST) with 5% non-fat milk and incubated for 1 h at 37 °C. After three washes, the membranes were incubated overnight at 4 °C using the following primary antibodies: anti-β-actin (Cat. No. 4970, Cell Signaling Technology (CST), MA, USA); anti-MRP1 (Cat. No. 14685S, CST); anti-MRP2 (Cat. No. 12559S, CST); anti-MRP3 (Cat. No. 14182S, CST); anti-GPAT3 (Cat. No. 71236S, CST); anti-STAT3 (Cat. No. 4904S, CST); anti-p-STAT3 (Tyr705) (Cat. No. 9145S, CST); anti-p-STAT3 (Tyr727) (Cat. No. 9134S, CST); anti-YY1 (Cat. No. 46395S, CST); anti-Bcl-2 (Cat. No. 3498S, CST); anti-Bcl-xL (Cat. No. 2762S, CST); anti-NF-κB p65 (Cat. No. 8242S, CST); anti-p-NF-κB p65 (Cat. No. 3033S, CST); anti-ATF3 (Cat. No. AF6240, Beyotime; anti-LC3A/B (Cat. No. 12741S, CST); anti-SQSTIM1/p62 (Cat. No. 39749S, CST); anti-GSDMD (Cat. No. 39754S, CST); anti-cleaved Caspase-3 (Cat. No. 9664S, CST). The membranes were then washed four times and incubated for 1 h at room temperature with a respective IgG-HRP labeled second antibody (Zhongshan Goldenbridge, Beijing, China) in TBST. Antigens were revealed using a chemiluminescence assay (Pierce, Rockford, USA) on a Bio-Rad system. Quantification of bands was achieved by densitometry using the AlphaView SA software.

### Treatment of cells with compounds, TAG or NaOA

TAG (50:0-FA18:0) was procured from Larodan (Cat. No. 34-1610-7) and dissolved in chloroform, while TAG (54:3-FA18:1) was obtained from Sigma-Aldrich (Cat. No. T7752). Sodium oleate (NaOA) was acquired from Meilunbio (Cat. No. MB2952-1). Following overnight cell seeding in well plates, TAG (50:0-FA18:0), TAG (54:3-FA18:1), and NaOA were diluted in 10% BSA without FFA in complete medium. BAY 11-7082 (Cat. No. HY-13453), ABT-199 (Cat. No. HY-15531), A922500 (Cat. No. HY-10038), PF-06424439 (Cat. No. HY-108341), and FSG67 (Cat. No. HY-112489) were purchased from MCE and dissolved in DMSO, while N-ethylmaleimide was obtained from MCE (Cat. No. HY-D0843) and dissolved in water. These chemicals were added to fresh medium at specific concentrations when utilized.

### Cell apoptosis assay

An Annexin V-FITC/PI Apoptosis Detection Kit (FXP018Pro, 4A Biotech) was utilized to detect cell apoptosis. The cells were initially seeded into 6-well plates. After treatment with sorafenib for the indicated time, MHCC97H cells were exposed to a concentration of 10 μM sorafenib, while Hep3B cells were exposed to 6 μM sorafenib. Samples were collected and processed according to the manufacturer's specifications. The apoptotic cells were subsequently analyzed using the NovaCyteAdvanteon Flow Cytometer (Agilent).

### Measurement of intracellular lipid droplets content by BODIPY 558/568 C12 staining

BODIPY (558/568 C12) was employed to label lipid droplets (LDs) in this study. Initially, cells were plated in a 6-well plate and incubated for 24 hours. Subsequently, the cells were exposed to sorafenib treatment for a specified period. The cells were then harvested and stained with BODIPY 558/568 C12 at 37°C for 15 minutes, followed by PBS washes, and lipid content quantification using FCs. For visualization purposes, cells were double-stained with Hoechst and BODIPY 558/568 C12 at 37°C for 30 minutes. After PBS washing, the samples were visualized using a confocal microscope (Leica).

### BODIPY 581/591 C11 staining for indexing lipid peroxidation

BODIPY (581/591 C11) was used to detect the levels of lipid peroxidation: cells were seeded in a 6-well plate and incubated for 24 h. After that, the cells were treated with or without sorafenib for the specified duration. Subsequently, the cells were collected and incubated with BODIPY 581/591 C11 at 37 °C for 15 min, washed with PBS, the fluorescence was detected by FCs.

### siRNA transfection assay

The siRNA targeting STAT3 (sequence: 5'-CGUCCAGUUCACUACUAAATT-3'), GPAT1 (1^#^ sequence: 5'-GGAAUGUUAUUUAUAUCAATT-3'; 2^#^ sequence: 5'-GAACAGCAGUAGAGUACAATT-3'; 3^#^ sequence: 5'-GGUCAACUUGAGAUGGUUATT-3'), GPAT4 (1^#^ sequence: 5'-GAGUAAACAUGUUCACUUATT-3'; 2^#^ sequence: 5'-CAUCGGUGAUGAUGUUCAATT-3'; 3^#^ sequence: 5'-GCACAACUGUGGUGGGAUATT-3'), were transfected into SR HCC cells by Lipofectamine^TM^3000 (Thermo Fisher) according to the manufacturer's instructions. After transfection for 24 h, qPCR and WB analysis were performed to evaluate the knockdown efficiency.

### UHPLC-MS/MS assay

Lipid analysis was conducted using an ultra-high performance liquid chromatography (UHPLC) I-Class system coupled with an AB Sciex Instruments 6500+ Q-TRAP tandem mass spectrometer (MS/MS) (Applied Biosystems, Foster City, CA, USA), as previously described 14. Before sample extraction, deuterated internal standards SPLASH® Lipidomix® (Cat. No. 330707, Avanti) were added. Samples were mixed with a chloroform-methanol mixture and acetone, dried, and eluted with a chloroform-isopropanol mixture. The extracted samples were analyzed using the Acquity UPLC system and the Sciex software. Table S5 provides details of the parameters and lipids used.

### ELISA assay

Cells were seeded in a 6-well plate and incubated for 24 hours. Subsequently, the levels of lysophosphatidic acid (LPA) in the cell supernatants were measured using the human LPA ELISA kit (Cat. No. YJ405877, mlbio). For the analysis of mouse liver LPA levels, 0.5 g of mouse liver tissue was homogenized in 3 ml of PBS, followed by centrifugation at 3000 rpm for 10 minutes to collect the supernatant. The LPA levels in both mouse plasma and liver tissue were then determined using the mouse LPA ELISA Kit (Cat. No. YJ323690, mlbio).

### Oil Red O staining

The tissues were fixed with 4% paraformaldehyde, dehydrated with 30% sucrose overnight, and sent to the Department of Pathology at Chongqing University Cancer Hospital for frozen sectioning. They were then stored at -20 °C. The frozen sections were reheated at room temperature for 10 min, fixed with 4% paraformaldehyde for another 10 min, and stained with oil red O (Cat. No. C0158, beyotime) staining reagent for 50 min. After cleaning with ice PBS, the sections were stained with hematoxylin for 2 min, followed by reverse blue staining with 1% hydrochloric alcohol for 10 s. The sections were then washed with tap water, sealed with anti-fluorescence quench agent, and observed and photographed under a microscope (BX53, Olympus). The mean density (IOD/area) of Oil Red O was detected in different positive areas of tissues using ImageJ software.

### Immunohistochemistry (IHC)

The xenograft was sectioned by the Oncology Laboratory at Chongqing University Cancer Hospital. Subsequently, the slides were stained using hematoxylin-eosin (H&E), MRP1, GPAT3, and NF-κB p65 antibody. The imaging was performed using a positive fluorescence microscope (BX53, Olympus). The mean density (IOD/area) of MRP1, GPAT3 and NF-κB p65 were detected in different positive areas of tissues using ImageJ software.

### Statistical analysis

The data were analyzed using two-tailed unpaired Student's t-test and one-way ANOVA with GraphPad Prism 8.0 (GraphPad Software, Inc., La Jolla, CA, USA) and Excel 2007 (Microsoft). Each experiment included a minimum of three independent samples. A p value less than 0.05 was considered statistically significant.

## Results

### Lipid metabolism participates in acquired sorafenib resistance of HCC

To investigate the relationship between lipid metabolism and HCC sorafenib resistance, we first established acquired sorafenib-resistant MHCC97H and Hep3B cells by continuously inducing them with a low concentration of sorafenib (Figure S1A). After being cultured for over a year, the sorafenib resistance group exhibited higher IC_50_ values against sorafenib compared to their parental group (Figure S1B). Additionally, the expression of multidrug resistance associated proteins (MRP), including MRP1, MRP2, MRP3, MRP4, MRP6, and multidrug resistance protein 1 (MDR1), noticeably increased in sorafenib-resistant cells compared to parental cells (Figure S1C-D). These results confirm the successful establishment of sorafenib-resistant (SR) HCC cells.

**Figure 1:**
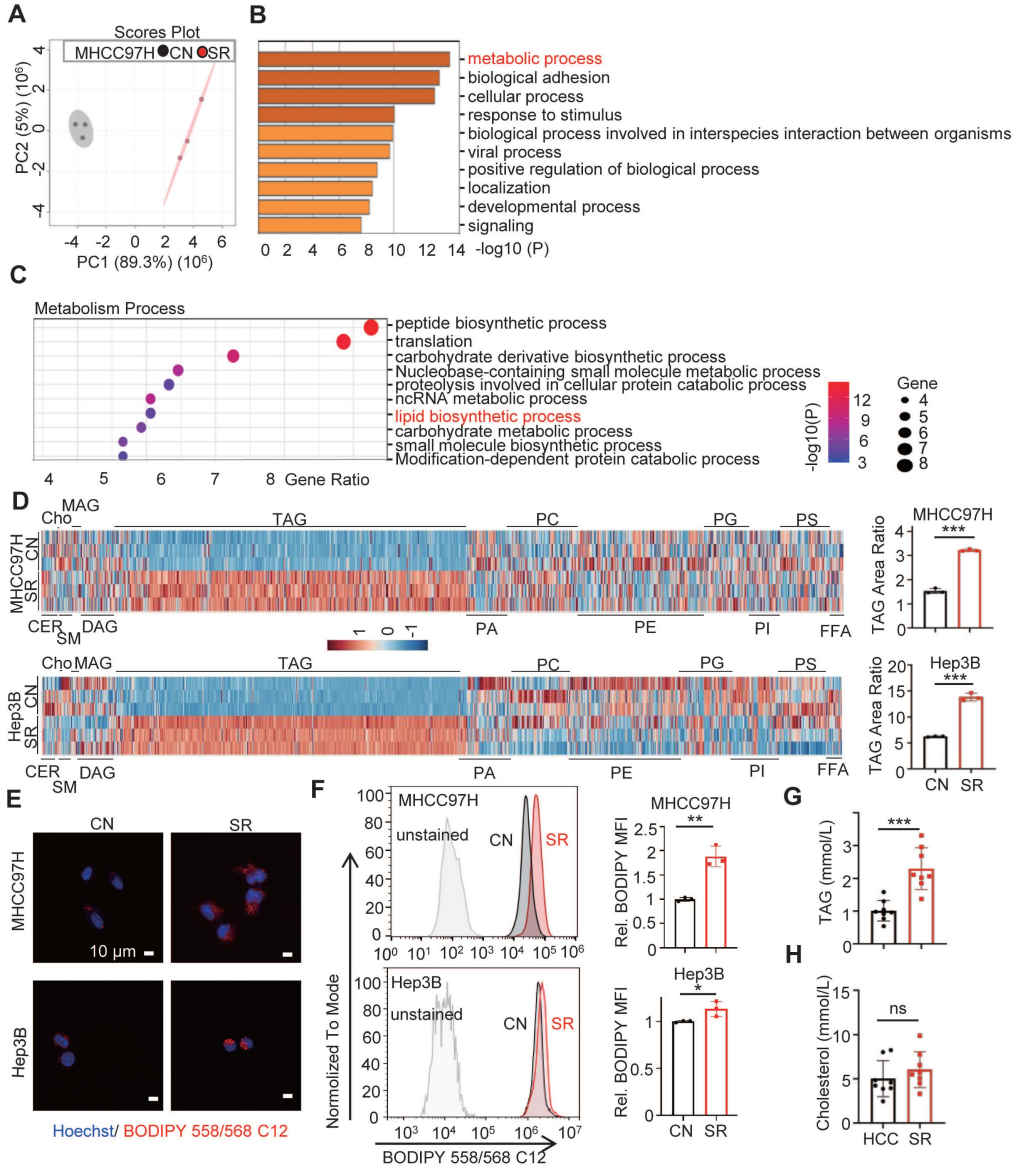
** Lipid metabolic reprogramming in SR HCC cells.** (**A**) Principal component analysis (PCA) of proteomic composition in parental (CN) and sorafenib resistant (SR) MHCC97H cells (n=3). (**B**) Gene Ontology (GO) analysis of differential expression genes in CN- and SR-MHCC97H cells. **(C)** Enrichment analysis of metabolism process in CN- and SR-MHCC97H cells. (**D**) Lipid contents in CN- and SR-MHCC97H and Hep3B cells were examined by UHPLC-MS/MS, and TAG area ratio of these cells was shown on the right (CER: ceramide; cho: cholesterol; SM: sphingomyelin; MAG: monoacylglycerol; DAG: diacylglycerol; TAG: triacylglycerol; PA: phosphatidic acid; PC: phosphatidylcholine; PE: phosphatidylethanolamine; PG: phosphoglyceride; PI: phosphatidylinositol; PS: phosphatidylserine; FFA: free fatty acid). (**E, F**) CN- and SR-MHCC97H and Hep3B cells were stained by Bodipy 558/568 C12, then determined by confocal microscopy and FCs. (**G**) TAG levels in the blood of clinical HCC patients before and after acquired SR. (**H**) Cholesterol levels in the blood of clinical patients before and after acquired SR. Data are expressed as means ± SEM (n = 3). *p < 0.05, **p< 0.01, ***p< 0.001. ns represents no significant difference.

Proteomic analysis was conducted and principal component analysis (PCA) revealed a clear separation between the MHCC97H cells in the parental group and the sorafenib resistance group (Figure 1A). Additionally, a significant difference in the metabolism process was observed between the two groups (Figure 1B). Specifically, the sorafenib resistance group exhibited a higher enrichment of lipid biosynthesis compared to the parental cells (Figure 1C). These findings indicate the involvement of lipid biosynthesis in sorafenib resistance of HCC.

### Triglyceride deposition is increased in sorafenib resistant HCC cells

To evaluate the intracellular lipid components in sorafenib-resistant hepatocellular carcinoma (SR HCC) cells, we utilized UHPLC-MS/MS assay to compare the differences in intracellular lipid components between SR HCC cells and their parental cells. Our findings revealed a significant increase in triacylglycerol (TAG) components in the SR-group cells compared to the control groups (Figure 1D, Figure S2, Table S6-7). While the contents of sphingomyelin and phospholipid were significantly reduced in SR-Hep3B cells compared to control Hep3B cells, this difference was not observed in MHCC7H cells. There were no noticeable differences in the components of ceramide, cholesterol, monoglyceride, and diglyceride between the SR-group and their parental cells. Consistent with these findings, we observed a higher level of lipid deposition in SR cells compared to the control (Figure 1E-F). The TAG area ratio increased by approximately 2.1 and 2.2 folds in the MHCC97H-SR and Hep3B-SR groups, respectively. Importantly, we discovered that TAG levels, but not cholesterol levels, were significantly higher in HCC patients after sorafenib resistance compared to those before sorafenib resistance (Figure 1G-H). These results indicate an increase in TAG levels in SR HCC cells.

### GPAT3 is highly expressed in sorafenib resistant HCC cells

**Figure 2:**
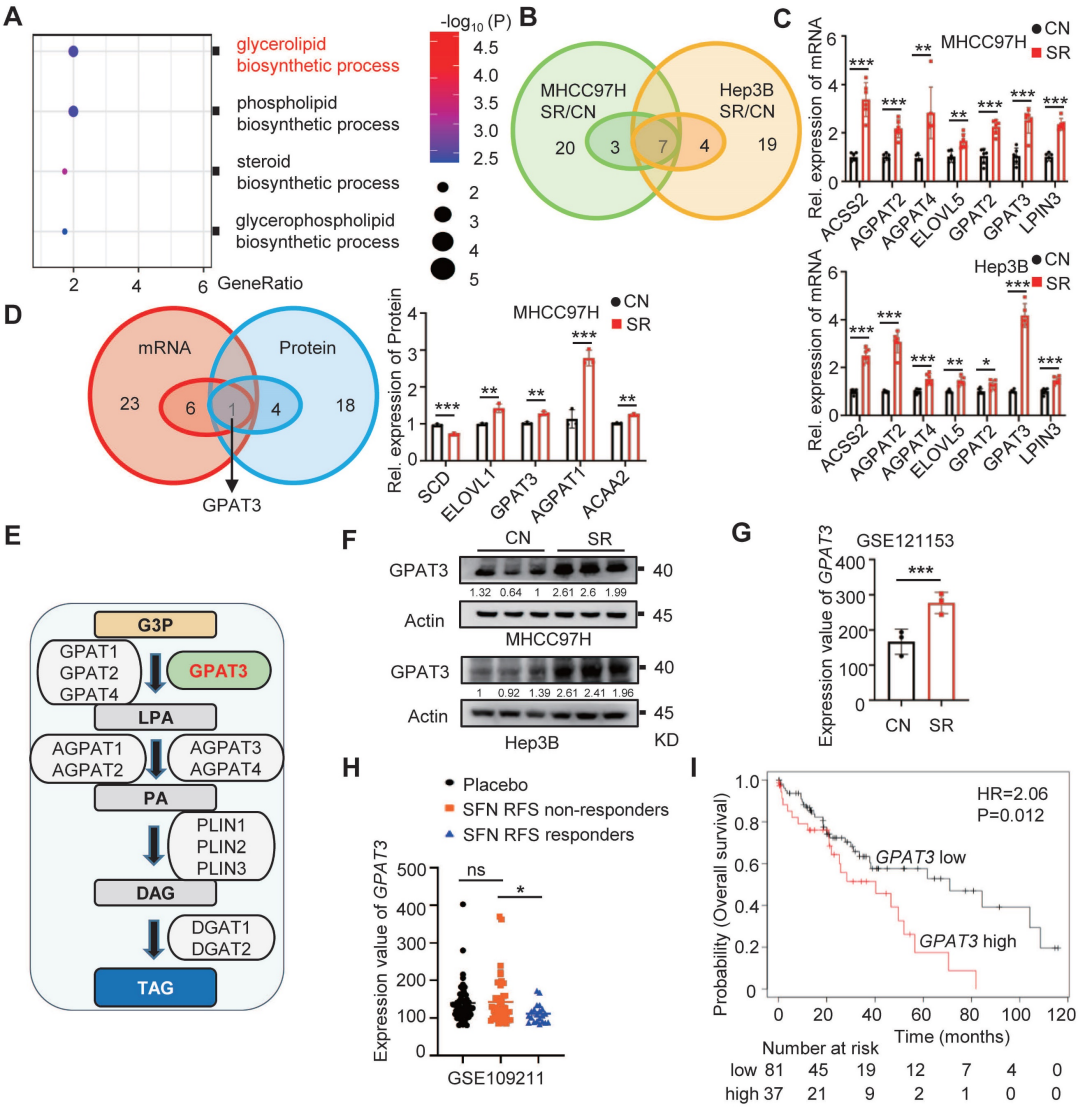
** GPAT3 is highly expressed in sorafenib resistance cells of HCC.** (**A**) Enrichment analysis of “lipid biosynthetic process” pathway in CN- and SR-MHCC97H cells. (**B**) *De novo* TAG synthesis-related mRNA expression in CN- and SR-MHCC97H and Hep3B cells. (**C**) Seven significantly differentially expressed mRNA genes related to *de novo* TAG synthesis were observed in CN-, SR-MHCC97H, and Hep3B cells. (**D**) *De novo* TAG synthesis-related mRNA and protein expression in CN- and SR-MHCC97H cells. (**E**) GPAT3 is involved in *de novo* synthesis pathway diagram of TAG. (**F**) Protein levels of GPAT3 in CN- and SR-MHCC97H and Hep3B cells were detected by WB. (**G**) Expression analysis of GPAT3 in parental and sorafenib-resistant cells xenograft tumor mouse model (GSE121153). (**H**) Expression of GPAT3 in HCC patients with or without RFS response treated with placebo or sorafenib (GSE109211). Data are expressed as means ± SEM (n = 3). (**I**) Kaplan Meier Plotter database analysis of prognosis in female patients with high or low GPAT3 expression. *p < 0.05, **p < 0.01, ***p < 0.001. ns represents no significant difference.

To investigate the cause of TAG accumulation in SR HCC cells, we conducted a detailed analysis of proteomic data. Our findings indicate that the glycerol lipid biosynthesis process was significantly more abundant in the SR group compared to parental cells (Figure 2A). Additionally, we examined a total of 30 genes and 23 proteins related to de novo TAG synthesis using PCR-array and proteomics data. Our analysis identified 7 up-regulated genes and 5 up-regulated proteins in the SR groups (Figure 2B-D, Figure S3A-B, Table S2-3). Through a comprehensive analysis, GPAT3 was pinpointed as a key factor in this process (Figure 2D).

GPAT3, a member of the lysophosphatidic acid acyltransferase protein family, has been identified as the primary rate-limiting enzyme in the TAG synthesis pathway (Figure 2E) 15. Our study revealed a 2~3 fold increase in GPAT3 protein levels in the SR group compared to control (Figure 2F). Analysis of the GEO database showed a 2-fold elevation in GPAT3 expression in xenografted tumor tissues with SR, but not in oxaliplatin and levantinib resistant HCC cells (Figure 2G, Figure S3C). Furthermore, patients with low GPAT3 levels exhibited extended recurrence free survival (RFS) when treated with sorafenib (Figure 2H). Additionally, a positive correlation was found between GPAT3 and multidrug resistance proteins gene such as ABCC2, ABCC6, and ABCC11, indicating a link between GPAT3 and drug resistance in HCC (Figure S4). Kaplan-Meier analysis based on microarray data from the Kaplan Meier Plotter database showed that high GPAT3 expression was associated with poor survival among HCC patients (Figure 2I). These results suggest that GPAT3 is overexpressed in SR HCC cells and is closely linked to the unfavorable prognosis of HCC patients.

### STAT3 is required for GPAT3 upregulation in SR HCC cells

**Figure 3:**
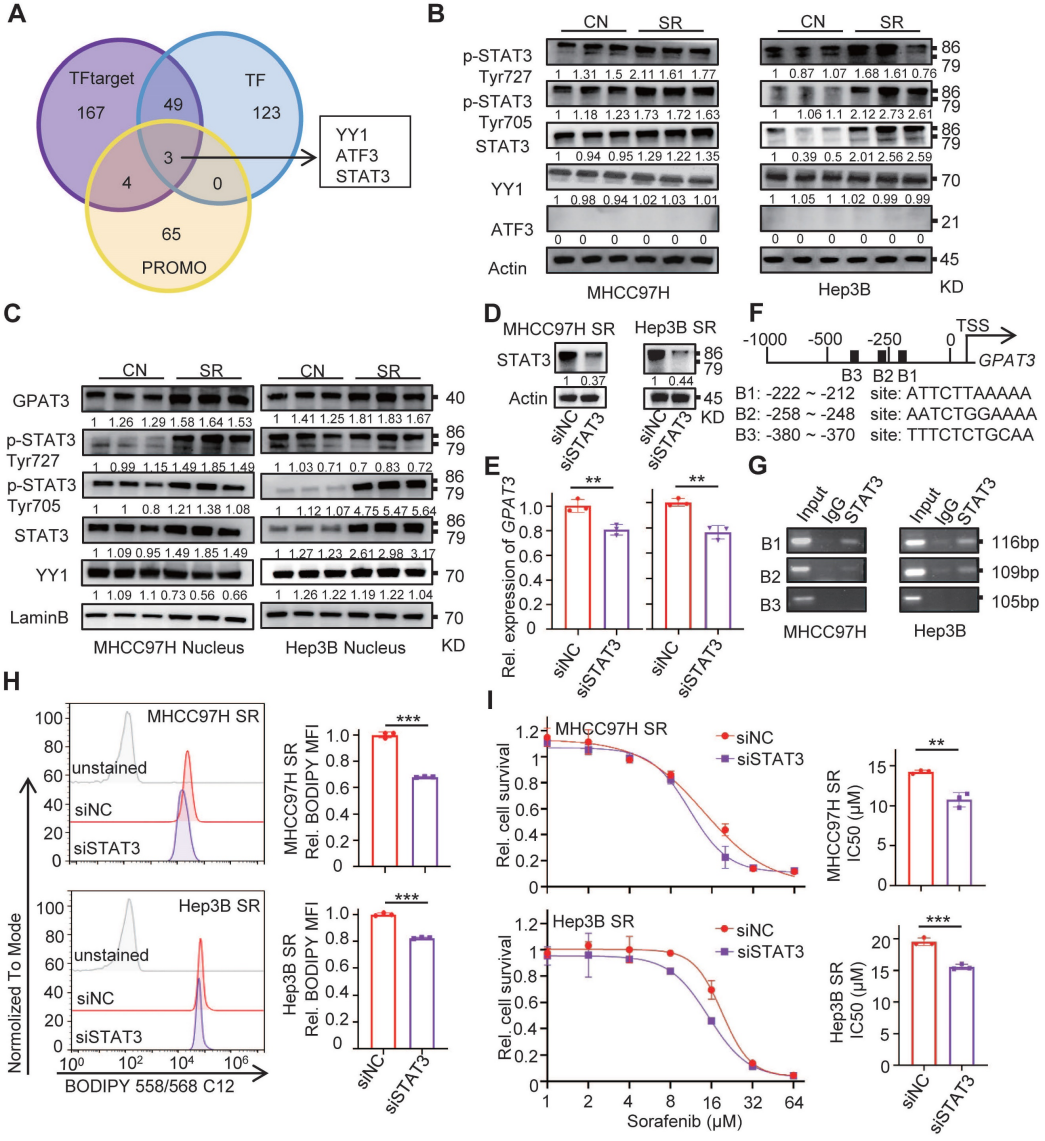
** GPAT3 is directly regulated by the transcription factor STAT3.** (**A**) Prediction of *GPAT3* promoter related transcription factors in TF target, TF and PROMO. (**B**) WB experiment to detect the expression of STAT3 and its phosphorylation of total protein in CN- and SR-MHCC97H and Hep3B cells. (**C**) WB was applied to detect the expression of STAT3 and its phosphorylation of nucleoprotein in CN- and SR-MHCC97H or Hep3B cells. (**D, E**) Protein levels of STAT3 (**D**), mRNA levels of GPAT3 (**E**) were examined after 24 h of siRNA transfection targeting STAT3. (**F, G**) Schematic diagram of binding sites between *GPAT3* promoter and STAT3 (**F**), ChIP experiment on STAT3 antibody binding to MHCC97H and Hep3B cells (**G**). (**H**) After 24 h of siRNA transfection targeting STAT3, MHCC97H and Hep3B-SR cells were stained with Bodipy 558/568 C12, and then measured by FCs. (**I**) Following 24 hours of siRNA transfection targeting STAT3, MHCC97H and Hep3B-SR cells were treated with varying concentrations (1, 2, 4, 8, 16, 32, 64 μM) of sorafenib for 72 hours. The IC_50_ was assessed using a CCK-8 assay. Data are expressed as means ± SEM (n = 3). *p < 0.05, **p < 0.01, ***p < 0.001.

To investigate the reasons for the upregulation of GPAT3 during SR, we utilized TF, TF target, and PROMO database to predict transcription factors at the human *GPAT3* promoter. Notably, the transcription factor Yin Yang 1 (YY1), activating transcription factor 3 (ATF3), and signal transducer and activator of transcription 3 (STAT3) were identified as potential regulators of GPAT3 transcription (Figure 3A).

Subsequently, we examined the levels of these 3 TFs in SR HCC cells and found that levels of STAT3 and its phosphorylated forms (p-Tyr727 and p-Tyr705) were significantly increased in SR HCC compared to the parental cells (Figure 3B). Additionally, there was a significant upregulation (~2-fold) of nuclear accumulation of STAT3 and its phosphorylated forms (p-Tyr727 and p-Tyr705) in SR cells (Figure 3C). However, no similar trend was observed for YY1 and ATF3 between SR cells and the parental cells.

STAT3, a member of the STAT family, plays a role in shaping distinct metabolic processes that regulate tumor progression and therapy resistance by transducing signals from metabolites, cytokines, growth factors, and their receptors 16. In order to investigate the transcriptional regulation of *GPAT3* by STAT3, siRNA targeting STAT3 was transfected into MHCC97H and Hep3B SR cells. The mRNA level of GPAT3 decreased 20~30% after silencing STAT3 in SR cells (Figure 3D-E). Furthermore, JASPAR online analysis identified three putative STAT3-binding sites at the *GPAT3* promoter (Figure 3F) 17. To confirm the transcriptional regulation of *GPAT3* by STAT3, chromatin immunoprecipitation (ChIP) assays were performed, revealing a specific and direct interaction between the STAT3 protein and the B1 and B2 sites within the *GPAT3* promoter in HCC cells (Figure 3G). Knockdown of STAT3 by siRNA significantly reduced intracellular TAG content and enhanced sensitivity to sorafenib in MHCC97H and Hep3B SR cells (Figure 3H-I). Collectively, these findings suggest that STAT3 is activated during SR and subsequently promotes *GPAT3* expression transcriptionally in HCC cells.

### GPAT3 enhances the insensitivity of HCC cells to sorafenib through increasing TAG accumulation

**Figure 4:**
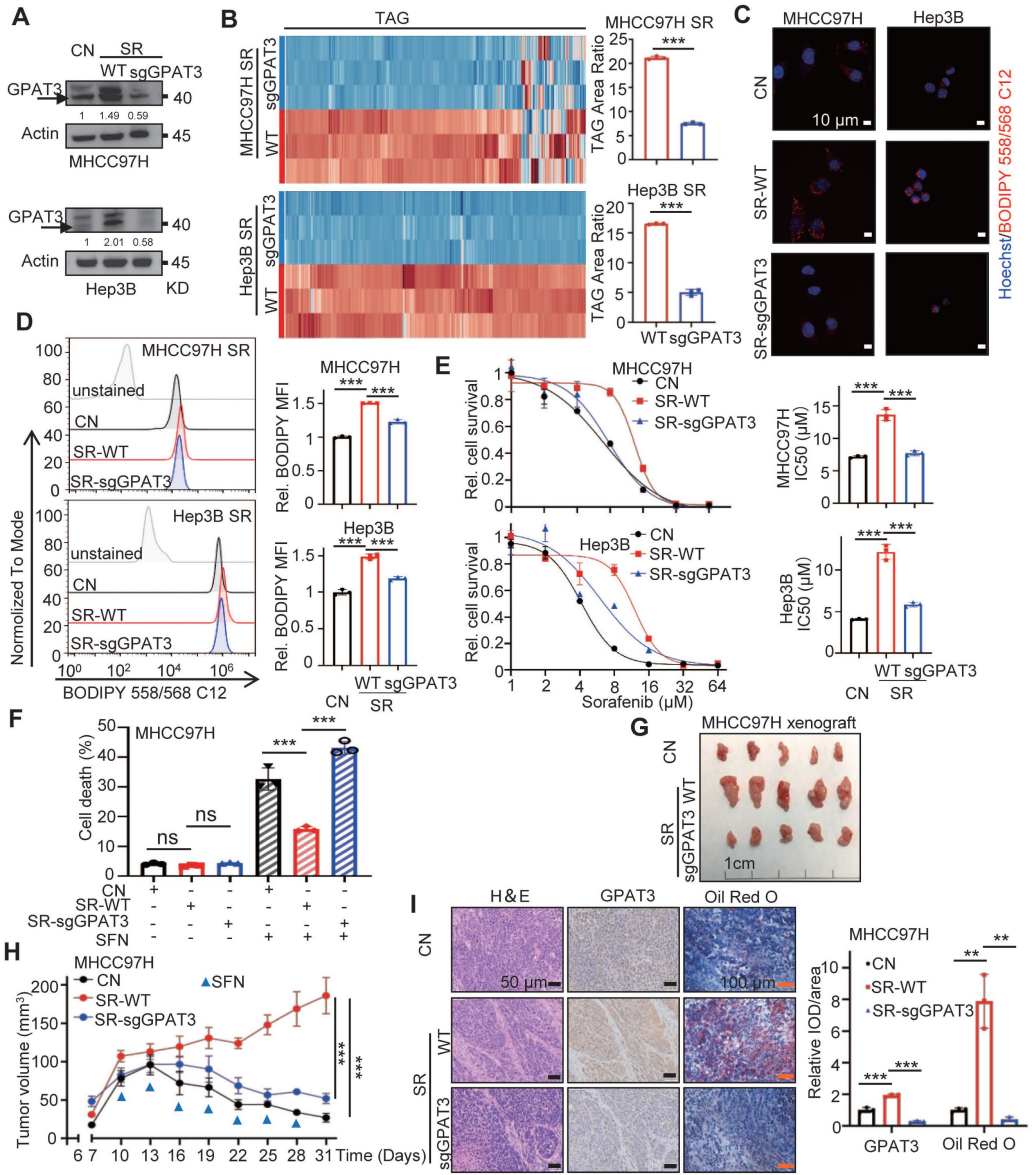
** GPAT3 deficiency reduces the lipid content and improves the sensitivity of SR cells to sorafenib.** (**A**) GPAT3 expressions in CN-, SR wildtype (SR-WT), SR GPAT3 knockdown (SR-sgGPAT3) and MHCC97H and Hep3B cells determined by WB. (**B**) TAG contents in SR-WT and SR-sgGPAT3 MHCC97H or Hep3B cells were examined by UHPLC-MS/MS, and TAG area ratio of these cells was shown on the right. (**C, D**) CN, SR-WT and SR-sgGPAT3 MHCC97H and Hep3B cells were stained by Bodipy 558/568 C12, then were determined by confocal microscopy (**C**) and FCs (**D**). Lipid droplets were represented in red, cell nucleus was labeled with DAPI (blue). (**E**) The IC_50_ values for CN, SR-WT, and SR-sgGPAT3 in MHCC97H and Hep3B cells were determined using the CCK-8 assay after treatment with sorafenib at various concentrations (1, 2, 4, 8, 16, 32, 64 μM) for 72 hours. (**F**) After treatment with/without sorafenib (MHCC97H: 10 μM; Hep3B: 6μM) for 72 h, cell death ratios of CN, SR-WT and SR-sgGPAT3 MHCC97H cells were determined by FCs. (**G**-**I**) Sensitivity of CN, SR-WT, SR-sgGPAT3 MHCC97H cells to sorafenib (2 mg/kg, once every 3 days) were evaluated *in vivo*. Tumor entity view (**G**), curves of tumor growth (**H**) and immunohistochemical (IHC) analysis for GPAT3 and Oil Red O staining (**I**) were shown. Data are expressed as means ± SEM (n = 3). *p < 0.05, **p < 0.01, ***p < 0.001. ns represents no significant difference.

To investigate the role of GPAT3 in sorafenib resistance in HCC, we utilized CRISPR-Cas9 technology to knockdown GPAT3 in SR cells (Figure 4A). The levels of TAG were measured using UHPLC-MS/MS and BODIPY fluorescence, and it was observed that GPAT3 knockdown led to a notable decrease (~2-3-fold reduction) in TAG levels without affecting LPA levels compared to control-SR cells (Figure 4B-D, Figure S5A-B, Table S8-9). This suggests that GPAT3 is required for TAG synthesis in SR cells. Furthermore, the knockdown of GPAT3 significantly improved the sensitivity of SR cells to sorafenib (Figure 4E). Besides, knockdown of DGATs or other GPATs in MHCC97H and Hep3B SR cells had no impact on TAG content or cell sensitivity to sorafenib (Figure S6A-H). Similarly, knocking down GPAT3 in parental Hep3B cells did not affect cell proliferation or sensitivity to sorafenib (Figure S5C-E). Additionally, we evaluated cell proliferation and death rates with or without sorafenib treatment after interfering with GPAT3. It is worth noting that knockdown of GPAT3 significantly amplified SFN-induced cell death in HCC SR cells, while had no significant effect on cell proliferation in SR HCC cell (Figure 4F, Figure S5F). These results suggested that elevated sensitivity to sorafenib induced by GPAT3 knockdown is mainly dependent on sorafenib-induced cell death.

To evaluate the potential clinical significance of targeting GPAT3 in HCC, a subcutaneous tumor model was established. In the absence of sorafenib treatment, the growth rate and tumor weight of SR xenografts remained unchanged following GPAT3 knockdown (Figure S5G-I). When treated with sorafenib, xenografts with GPAT3 knockdown exhibited enhanced sensitivity to sorafenib compared to the control SR-MHCC97H group (Figure 4G-H). Immunohistochemistry (IHC) assays revealed increased expression of GPAT3 and higher lipid deposition in SR xenografts relative to the control group. Furthermore, GPAT3 deficiency significantly reduced the lipid content in SR transplanted tumors (Figure 4I). These findings collectively indicate that GPAT3 plays a crucial role in SR both *in vitro* and *in vivo*.

**Figure 5:**
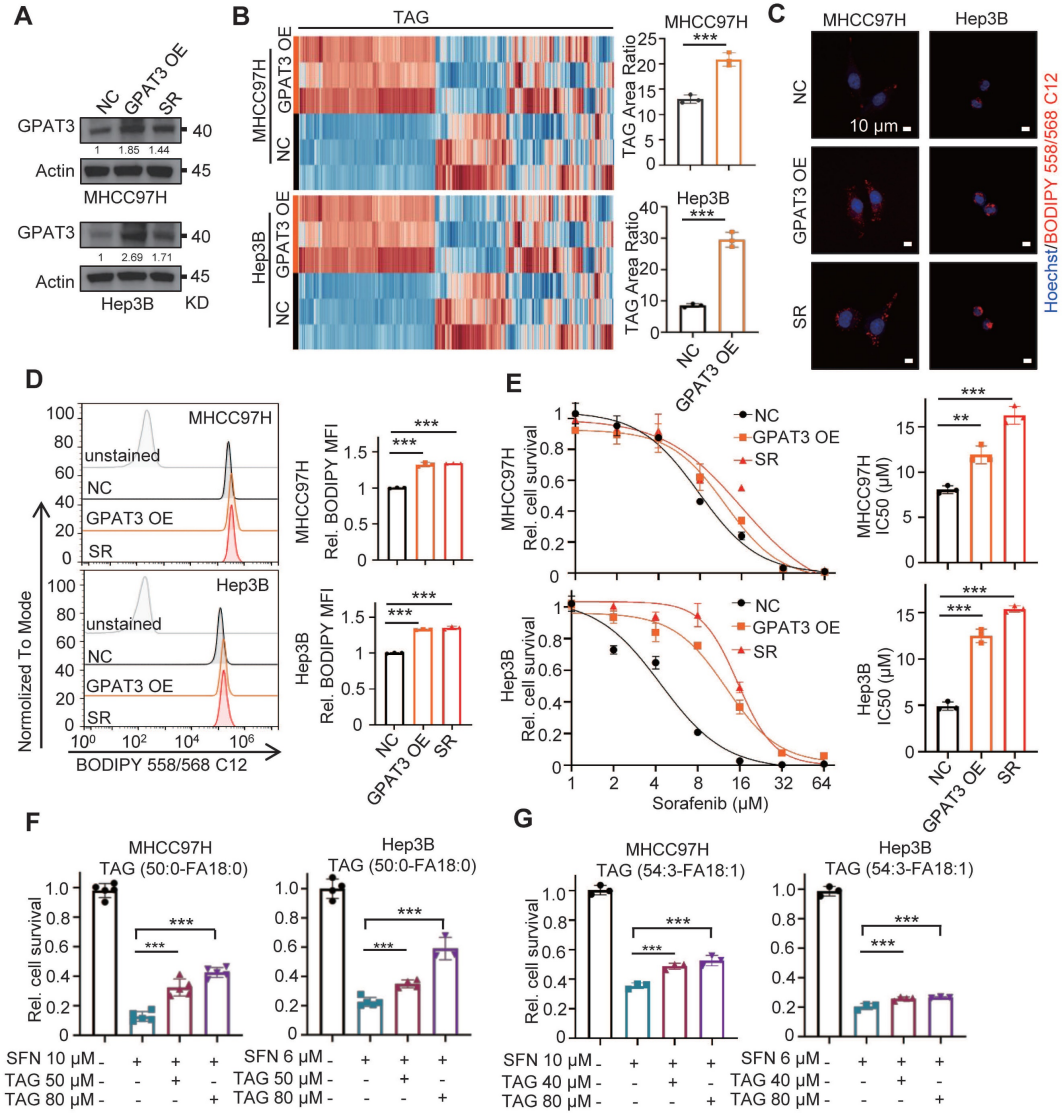
** Overexpressing GPAT3 increasing TAG accumulation and enhances the insensitivity of HCC cells to sorafenib.** (**A**) GPAT3 expressions in negative control (NC), GPAT3 overexpression (OE) and sorafenib resistance (SR) MHCC97H and Hep3B cells determined by WB. (**B**) TAG contents in NC- and GPAT3 OE-MHCC97H or Hep3B cells were examined by UHPLC-MS/MS, and TAG area ratio of these cells was shown. (**C, D**) NC-, GPAT3 OE- and SR- MHCC97H and Hep3B cells were stained by Bodipy 558/568 C12, then determined by fluorescence microscope (**C**) and FCs (**D**). Lipid droplets were represented in red, cell nucleus was labeled with DAPI (blue). (**E**) IC_50_ values were determined for NC-, GPAT3 OE-, and SR- MHCC97H and Hep3B cells using the CCK-8 assay after treatment with varying concentrations (1, 2, 4, 8, 16, 32, 64 μM) of sorafenib for 72 hours. (**F**,** G**) CCK-8 was used to detect cell survival of MHCC97H cells treated with sorafenib (10 μM) and Hep3B cells treated with sorafenib (6 μM) in combination with varying concentrations of TAG (50:0-FA 18:0 or 54:3-FA 18:1). Data are expressed as means ± SEM (n = 3). **p < 0.01, ***p < 0.001.

To further validate the role of GPAT3 in HCC cells, we conducted an experiment where we overexpressed GPAT3 in MHCC97H and Hep3B cells using a lentiviral vector (Figure 5A). Our findings showed a significant increase (~2-3 -fold up-regulation) in TAG levels after the introduction of GPAT3 (Figure 5B-D, Figure S7, Table S10-11). Additionally, the overexpression of GPAT3 was found to promote resistance to sorafenib in HCC cells (Figure 5E). Furthermore, the administration of TAG (50:0-FA 18:0 or 54:3-FA 18:1) was able to dose-dependently reverse the cell death caused by sorafenib in Hep3B and MHCC97H cells (Figure 5F-G). Taken together, these findings suggest that GPAT3 facilitates sorafenib resistance in HCC by increasing the accumulation of intracellular TAG.

### GPAT3 inhibits apoptosis of HCC cells by activating TAG-mediated NF-κB/Bcl2 signaling pathway

**Figure 6:**
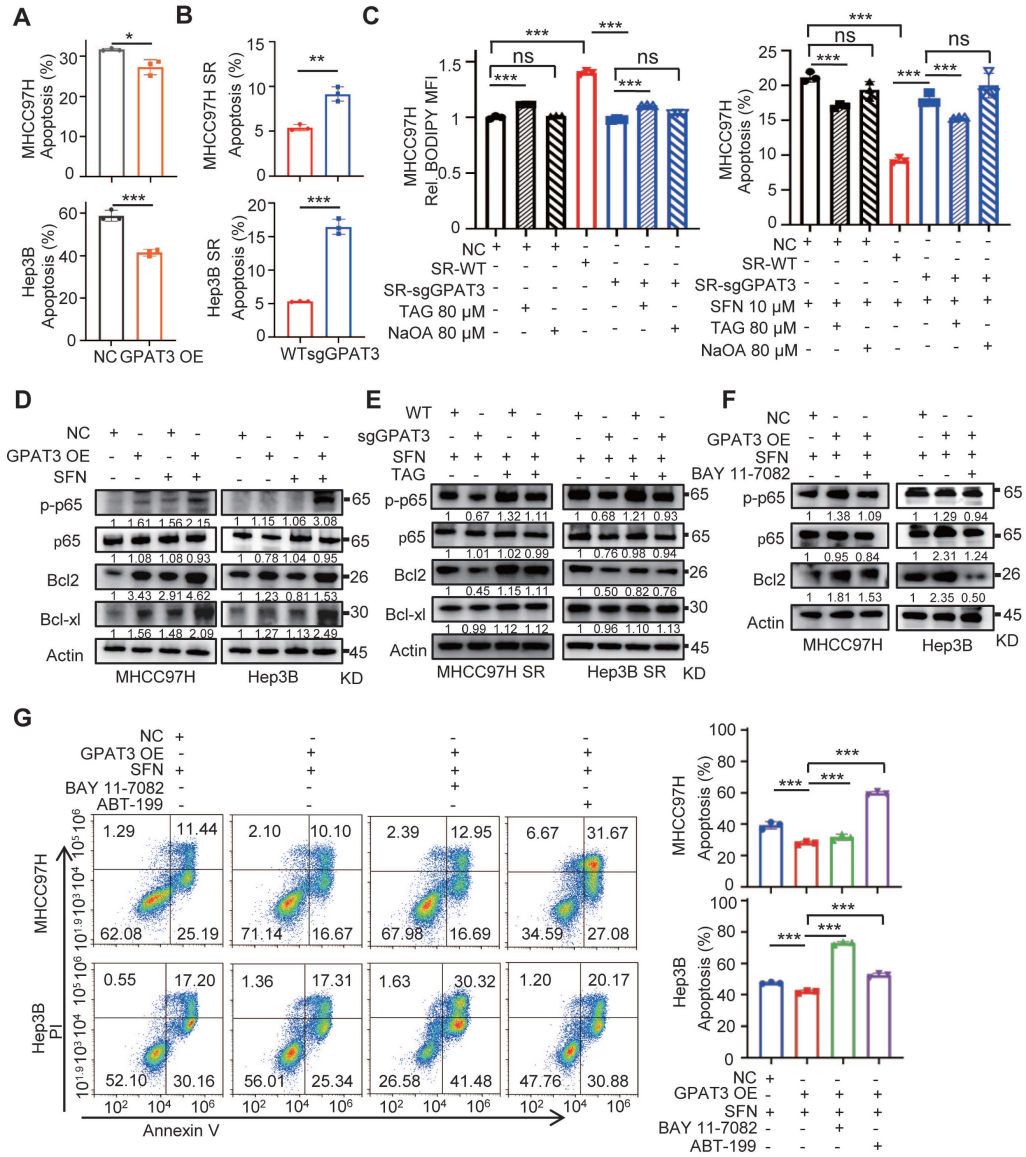
** GPAT3 inhibits apoptosis of HCC cells by TAG-initiated p65/BCL2 signaling pathway.** (**A, B**) After treatment with sorafenib (MHCC97H: 10 μM; Hep3B: 6μM) for 72h, cell apoptosis ratios of NC- and GPAT3 OE- MHCC97H or Hep3B cells (**A**), WT- and sgGPAT3- MHCC97H SR or Hep3B SR cells (**B**) were determined by FCs. (**C**) CN, SR-WT, and SR-sgGPAT3 MHCC97H cells were treated with 80 μM TAG (54:3-FA 18:1) or NaOA (80 μM), followed by staining with Bodipy 558/568 C12 and analysis using flow cytometry. The apoptosis ratios of MHCC97H cells treated with sorafenib (10 μM) in combination with 80 μM TAG (54:3-FA 18:1) or NaOA were also determined using flow cytometry. (**D**) NC- and GPAT3 OE- MHCC97H and Hep3B cells were treated with or without sorafenib (MHCC97H: 10 μM; Hep3B: 6μM) for 72 h. The total cell lysates were extracted and subjected to WB with indicated antibodies. (**E**) WT- and sgGPAT3- MHCC97H SR and Hep3B SR cells were treated with sorafenib (MHCC97H: 10 μM; Hep3B: 6μM) and TAG for 72 h. The total cell lysates were extracted and subjected to WB with indicated antibodies. (**F**) NC- and GPAT3 OE- MHCC97H and Hep3B cells were treated with sorafenib (MHCC97H: 10 μM; Hep3B: 6μM) for 72 h, combined with or without BAY 11-7082 (10 μM), the total cell lysates were extracted and subjected to WB with indicated antibodies. (**G**) NC- and GPAT3 OE- MHCC97H and Hep3B cells were treated with sorafenib (MHCC97H: 10 μM; Hep3B: 6μM), with/without BAY 11-7082 (3 μM) or ABT-199 (3 μM) for 72 h. Cell apoptosis ratios were determined by FCs. Data are expressed as means ± SEM (n = 3). **p < 0.01, ***p < 0.001. ns represents no significant difference.

In order to further understand how GPAT3 regulates sorafenib resistance in HCC, we conducted additional investigations. We assessed various types of cell death, such as apoptosis, autophagy, necrosis, and ferroptosis, and their impact on cell sensitivity to sorafenib. The results showed a notable decrease in the number of apoptotic cells when GPAT3 was overexpressed during sorafenib treatment. However, no significant differences were observed in the ratios of autophagy, necrosis, and ferroptosis. (Figure 6A, Figure S8A-C). Conversely, when GPAT3 was knocked down, ~2-3 -fold higher apoptosis ratio was detected (Figure 6B, Figure S8D). Supplementation of TAG slightly upregulated BODIPY levels and mildly attenuated sorafenib-induced apoptosis in CN and SR-sgGPAT3 MHCC97H cells. These effects were notably lower compared to MHCC97H SR cells, which exhibit higher GPAT3 expression. Additionally, the study found that NaOA treatment did not elevate TAG levels in CN and SR cells or impact cell sensitivity to sorafenib-induced apoptosis (Figure 6C). These findings suggest that the sorafenib insensitivity of SR HCC cells primarily stems from GPAT3-mediated *de novo* biosynthesis of TAG rather than the exogenous uptake of TAG or FFA.

NF-*κ*B is well known for providing cancer cells with a survival advantage by upregulating antiapoptotic genes, such as the Bcl2 family 18, 19. The interplay between NF-*κ*B and lipid metabolism has also been confirmed 20. Therefore, we investigated the effect of overexpressing GPAT3 on the level of anti-apoptotic proteins. Our results showed that overexpression of GPAT3 activated p-NF-κB p65 and upregulated the levels of Bcl2 and Bcl-XL proteins (~2 folds) under sorafeinb treatment (Figure 6D). Additionally, supplementing TAG significantly increased the levels of p65 and Bcl2 in GPAT3-null SR cells (Figure 6E). Furthermore, we found that the NF-κB inhibitor noticeably reduced Bcl2 protein levels in GPAT3-overexpressing HCC cells (Figure 6F). Importantly, when both NF-*κ*B and Bcl2 inhibitors were added, the apoptotic resistance triggered by overexpressing GPAT3 in HCC cells was abolished (Figure 6G). These findings suggest that GPAT3 inhibits cell apoptosis and promotes the survival of HCC cells by activating the TAG-mediated NF-*κ*B/Bcl2 signaling pathway.

### Pan-GPAT inhibitor reverses the sorafenib resistance of HCC cells *in vitro* and *in vivo*

**Figure 7:**
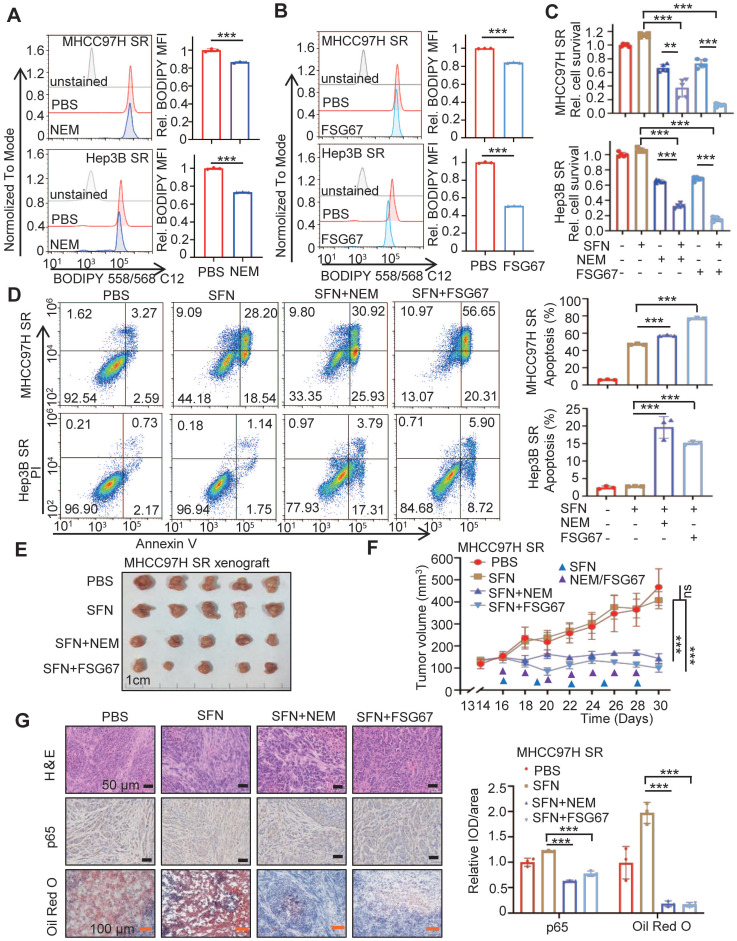
** GPAT3 inhibitor reverses the sorafenib resistance of HCC.** (**A**, **B**) After treatment with N-Ethylmaleimide (10 μM) (**A**) or FSG67 (30 μM) (**B**) for 24 h, MFI of Bodipy 558/568 C12 in MHCC97H SR and Hep3B SR cells were examined by FCs. (**C, D**) MHCC97H SR and Hep3B SR cells were treated with sorafenib (MHCC97H SR: 10 μM; Hep3B SR: 6μM) for 24 h, combined with or without NEM (10 μM) or FSG67 (30 μM). Cell deaths were estimated by CCK-8 assay (**C**) and apoptosis were estimated by FCs (**D**). (**E**-**G**) Sensitivity of MHCC97H SR cells to sorafenib (2 mg/kg, once every 3 days), NEM (1 mg/kg, once every 2 days) and FSG67 (1 mg/kg, once every 2 days) were evaluated *in vivo*. Tumor entity view (**E**), curves of tumor growth (**F**) and immunohistochemical (IHC) analysis for p65 and Oil Red O staining (**G**) were displayed, and statistical analysis of IOD of p65, Oil red O staining (right panel). Data are expressed as means ± SEM (n = 3). *p < 0.05, **p < 0.01, ***p < 0.001. ns represents no significant difference.

We then investigated whether blocking GPAT3 could enhance the sorafenib sensitivity of HCC. Consistent with previous findings, the inhibition of GPAT activity using two pan-GPAT inhibitors, N-ethylmaleimide (NEM) 21 and FSG67 22, significantly decreased intracellular TAG levels and improved apoptosis and sorafenib sensitivity in SR HCC cells (Figure 7A-D). Additionally, we utilized the MHCC97H subcutaneous tumor-bearing model to assess whether GPAT inhibitors could enhance SR *in vivo*. The GPAT inhibitor significantly decreased LPA levels in the mice liver and enhanced the sensitivity of SR xenografts to sorafenib. Interestingly, there was no impact on mouse weight or LPA levels in plasma (Figure 7E-F, Figure S9A-C). Furthermore, the IHC assay demonstrated that both pan-GPAT inhibitors significantly reduced lipid content and NF-*κ*B p65 expression in SR xenografts (Figure 7G). These findings suggest that blocking GPAT3, represents a promising strategy to enhance sorafenib response in HCC.

## Discussion

Sorafenib, a first-line targeted drug for advanced liver cancer, has limited application due to acquired resistance 5. In this study, we observed a significant increase in TAG accumulation in SR HCC cells. Through multi-omics analysis, we identified upregulated GPAT3 as the key enzyme involved in sorafenib resistance. Transcriptional activation of *GPAT3* in SR is mediated by STAT3, which directly binds to the *GPAT3* promoter. Loss- and gain-of-function experiments demonstrated that GPAT3 promotes sorafenib resistance in HCC by enhancing TAG-mediated NF-κB/Bcl2 signaling pathway. Additionally, pan-GPAT inhibitors restored sorafenib sensitivity both *in vitro* and *in vivo*. These findings uncovered a novel molecular basis by which lipid metabolic reprogramming mediates sorafenib resistance of HCC.

Previous studies have already discussed the impact of various lipids on SR in HCC. For example, inhibiting sphingolipid metabolism by increasing ceramide levels and decreasing sphingomyelin levels leads to mitochondrial dysfunction, oxidative stress, and enhances the sensitivity of resistant cells to sorafenib 23. Additionally, the inclusion of phosphatidylcholine and cholesterol alters the physical and chemical properties of the cell membrane, disrupting the phospholipid bilayer and thereby influencing sorafenib sensitivity 24, 25. Furthermore, cholesterol metabolism and the ratio of MUFA/PUFA also play a role in regulating sorafenib resistance in HCC 26. In our current study, we observed a significant accumulation of TAG in the sorafenib resistant group. Subsequently, supplementing TAG in normal HCC cells prevented cell death caused by sorafenib. Conversely, reducing TAG synthesis in SR HCC cells improved the sensitivity of these cells to sorafenib. However, considering that no significant upregulation of other enzymes in TAG synthesis in the SR cells model, blocking these enzymes may also bring more side effects. Sorafenib, a multi-kinase inhibitor, has been shown to have pro-apoptosis, anti-proliferation, and anti-angiogenesis effects by inhibiting various kinases including vascular endothelial growth factor receptor (VEGFR) 2, platelet-derived growth factor receptor (PDGFR-β), hepatocyte factor receptor (c-Kit), FMS-like tyrosine kinase 3 (FLT3), and intracellular Raf family kinase (4, 5). Previous studies have highlighted the issue of antiangiogenic drug (AAD) resistance in cancer patients, and have suggested that lipid-dependent metabolic reprogramming may be an alternative mechanism for AAD resistance. Additionally, targeting both angiogenesis and FAO has shown to increase the anti-tumor activity 27. In this study, our focus was to investigate the effect of sorafenib on the reprogramming of signaling pathways within HCC cells. Our SR HCC model was constructed *in vitro*, and omics data revealed significant changes in lipid metabolism pathways, while no significant changes were observed in angiogenic signals (such as VEGF, PDGF, FGF, *etc*). In addition, the expression of GPAT3 and TAG accumulation were detected in SR-resistant xenografts during the *in vivo* study. Furthermore, consistent results were obtained in both *in vitro* and *in vivo* experiments after knocking out GPAT3. No significant changes in angiogenic features were observed in GPAT3 knockout xenograft models. These findings suggest that the role of GPAT3 in facilitating SR maybe independent of its pro-angiogenesis effect. Besides, the sorafenib insensitivity of SR HCC cells, which possess higher GPAT3 expresion, is primarily dependent on de novo biosynthesis of TAG rather than exogenous uptake of TAG.

Induction of apoptosis is a well-known pathway of cytotoxicity for many chemotherapeutic drugs, including sorafenib 28. However, the tolerance to apoptosis is a crucial factor contributing to sorafenib resistance in HCC 5. In this study, we discovered that the transcriptional activation of *GPAT3* was mediated by STAT3. Moreover, GPAT3-mediated lipid metabolic reprogramming played a role in promoting apoptotic tolerance by activating the NF-κB/Bcl2 signaling pathway. STAT3 has been recognized as a target for inducing apoptosis in various tumors 29, 30. Additionally, the NF-κB p65 signaling pathway is a well-established anti-apoptotic pathway, primarily achieved by inducing and upregulating the expression of anti-apoptotic genes 31. Therefore, our data identified a novel signaling cascade, involving STAT3-GPAT3-TAG-NF-κB/Bcl2 signaling, which regulates apoptosis.

The crucial role of GPAT3 in TAG synthesis is widely recognized 32. Researchers have discovered that inhibiting DGAT can decrease TAG levels in HepG2 cells 33. In this study, we observed that after interference with DGATs or other GPATs in HCC SR cells were not observed triglyceride levels change and their sensitivity to sorafenib influence, but knocking down GPAT3 resulted in reduced TAG synthesis and increased apoptosis of HCC cells induced by sorafenib. As there is currently no commercially available specific inhibitor for GPAT3, we utilized NEM and FSG67 to inhibit the activity of GPAT isoforms. Remarkably, we also observed a decrease in TAG levels in SR HCC cells after inhibitor treatment.

The study has several unresolved issues that require further investigation. Firstly, due to time constraints and the unavailability of GPAT3 inhibitors, the clinical relevance was not thoroughly explored. Secondly, although GPAT3 also regulates phospholipids, the present study did not investigate the role of phospholipids in sorafenib resistance. Thirdly, the vascular in the tumor microenvironment was not investigated in this study, which is essential for the bioaction of sorafenib. Moreover, further exploration is needed to determine whether GPAT3 also plays a role in regulating resistance to other anti-angiogenic agents and immune checkpoint inhibitors.

Enhancing the sensitivity of HCC cells to sorafenib is of utmost importance for targeted therapy in clinical settings 2, 4. In this study, we have made a significant finding that STAT3 activates the transcription of *GPAT3* in SR HCC cells. This activation leads to the accumulation of TAG and subsequently activates the NF-κB/Bcl2 signaling pathway. These molecular events contribute to the development of apoptosis tolerance and the acquisition of sorafenib resistance. Notably, we have also discovered that inhibitors targeting pan-GPAT effectively enhance the sensitivity of SR HCC cells to sorafenib, both *in vitro* and *in vivo*.

## Conclusions

This preclinical investigation establishes a solid molecular foundation for understanding how GPAT3 promotes the acquisition of sorafenib resistance through lipid metabolism reprogramming. Furthermore, our findings suggest that GPAT3 could serve as a promising target for overcoming clinical resistance to sorafenib.

## Supplementary Material

Supplementary figures.

Supplementary tables.
