# Supplementary material for: GPAT3 is a potential therapeutic target to overcome sorafenib resistance in hepatocellular carcinoma
Source: Theranostics. 2024 Jun 1;14(9):3470–85. doi: 10.7150/thno.92646 (PMC11209725; doi:10.7150/thno.92646)
Supplement: Supplementary file 1 — Supplementary figures. [file thnov14p3470s1.pdf]

Supplementary information

Number of supplementary figures = 9, number of supplementary tables = 11.

Supplementary Figures

Figure S1

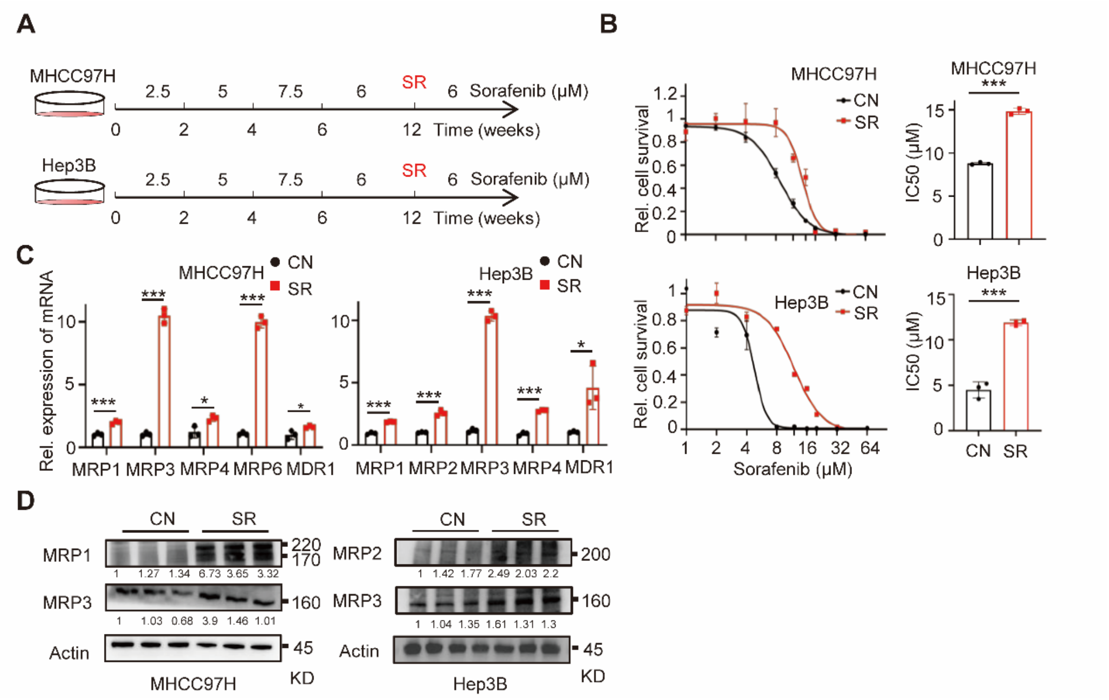

Figure S1. Establishment of acquired sorafenib resistant HCC cells by continuous induction with sorafenib. (**A**) Schematic models of induction of acquired sorafenib resistant (SR) HCC cells. (**B**) IC_{50} for sorafenib of CN- and SR- MHCC97H or Hep3B cells were evaluated by CCK-8 assay after different concentrations (1, 2, 4, 8, 16, 32, 64 μM) of sorafenib treatment for 72 h. (**C**) MRP1, MRP2, MRP3, MRP4, MRP6 and MDR1 mRNA expression in CN and SR- MHCC97H and Hep3B cells were determined by RT-qPCR. (**D**) Protein levels of MRP1 or MRP2 and MPR3 in CN- and SR- MHCC97H and Hep3B cells were determined by RT-qPCR. Data are expressed as means ± SEM (n = 3). *$p < 0.05$, **$p < 0.01$, ***$p < 0.001$.

Figure S2

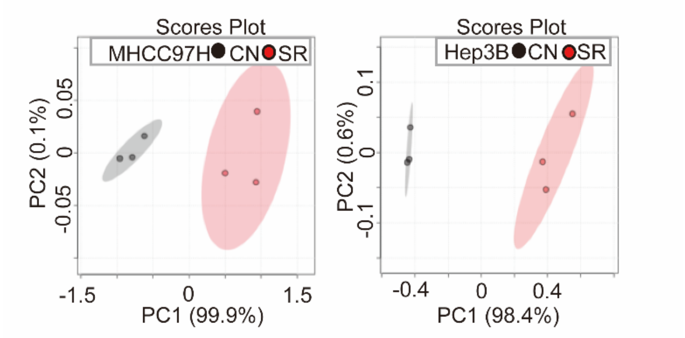

Figure S2. Principal component analysis (PCA) of UHPLC-MS/MS composition in CN- and SR- MHCC97H or Hep3B cells (n=3).

Figure S3

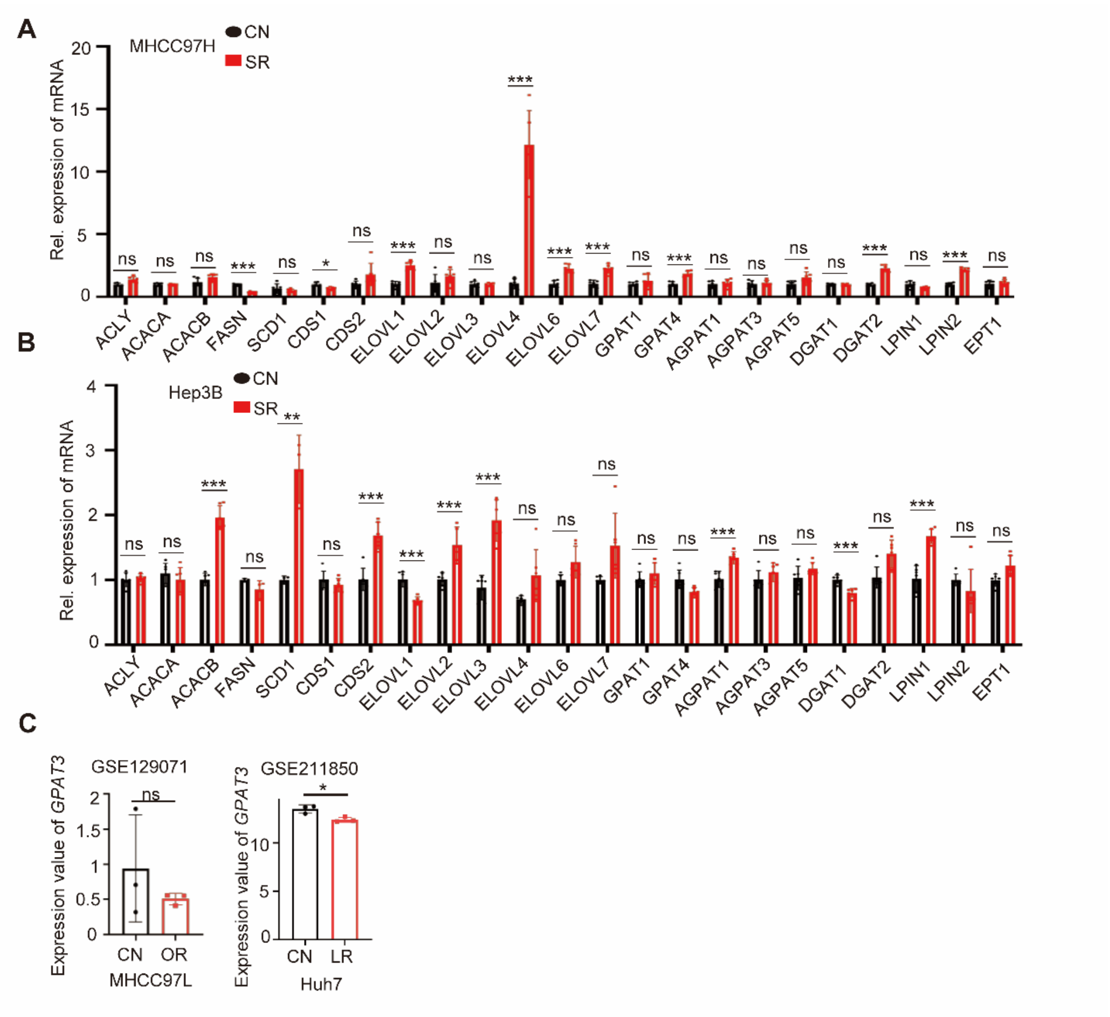

Figure S3. Enzymes involved in TAG synthesis expressions in SR HCC cells. (**A-B**) mRNA levels of Enzymes involved in TAG synthesis in CN- and SR- MHCC97(**A**) and Hep3B(**B**) cells were examined by qPCR. (**C**) Analysis of GPAT3 expression in parental and oxaliplatin resistant MHCC97L cells (GSE129071), lenvatinib resistant Huh7 cells (GSE211850)**.** Data are expressed as means ± SEM (n = 3). *$p < 0.05$, **$p < 0.01$, ***$p < 0.001$, ns represents no significant difference.

Figure S4

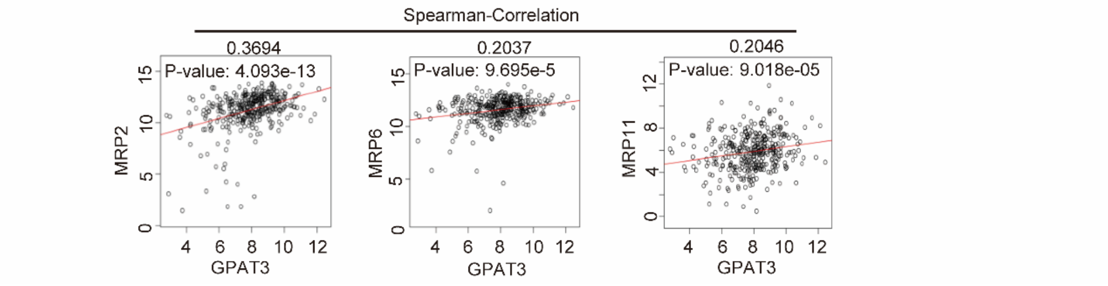

Figure S4. Linear correlation analysis between expression of GPAT3 and *ABCC2*, *ABCC6* and *ABCC11* expression in tumor tissues of HCC patients, data were obtained from TCGA database.

Figure S5

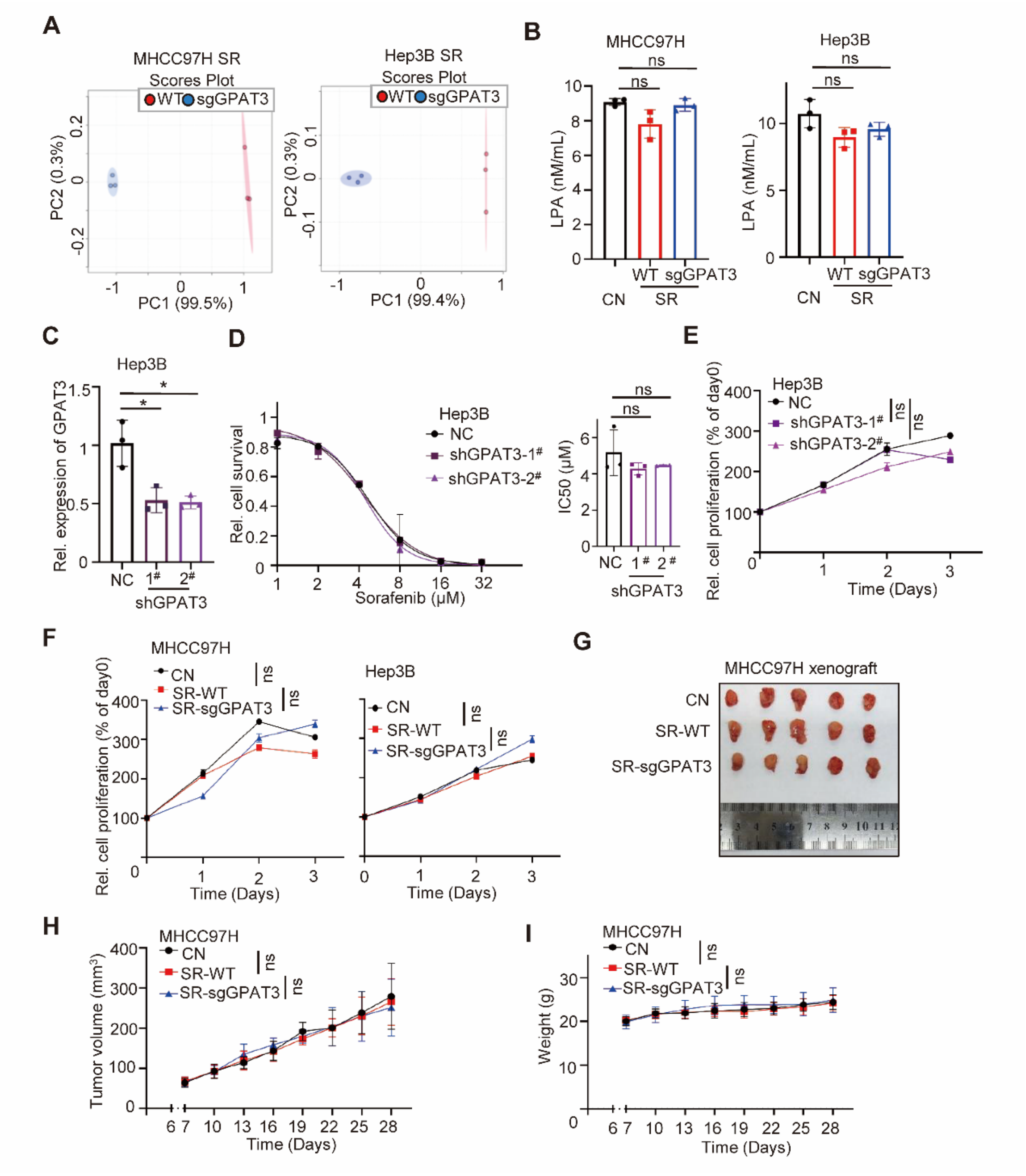

Figure S5. The effects of knockdown of GPAT3 on the proliferation of CN- and SR- HCC cells *in vitro* and *in vivo*. (**A**) PCA of TAG composition determined by UHPLC-MS/MS in WT- and sgGPAT3- MHCC97H SR or Hep3B SR cells (n=3). (**B**) LPA levels in CN-, SR-WT and SR-sgGPAT3 in MHCC97H or Hep3B cells were examined by ELISA (n=3). (**C**) GPAT3 levels in NC- and shGPAT3- Hep3B cells were examined by qPCR. (n=3). (**D**) The IC_{50} values for sorafenib in NC- and shGPAT3- Hep3B cells were determined using a CCK-8 assay following treatment with various concentrations (1, 2, 4, 8, 16, 32

μM) of sorafenib for 72 hours. (**E**) Cell proliferation of NC- and shGPAT3 Hep3B cells were evaluated by CCK-8 assay. (**F**) Cell proliferation of CN, SR-WT and SR-sgGPAT3 in MHCC97H or Hep3B cells were evaluated by CCK-8 assay. (**G-I**) Curves of tumor growth (**H**), tumor entity view (**G**) and weight change curve (**I**) in group of CN, SR-WT and SR-sgGPAT3 in MHCC97H cells without any treatment. Data are expressed as means ± SEM (n = 3). *$p < 0.05$, **$p < 0.01$, ns represents no significant difference.

Figure S6

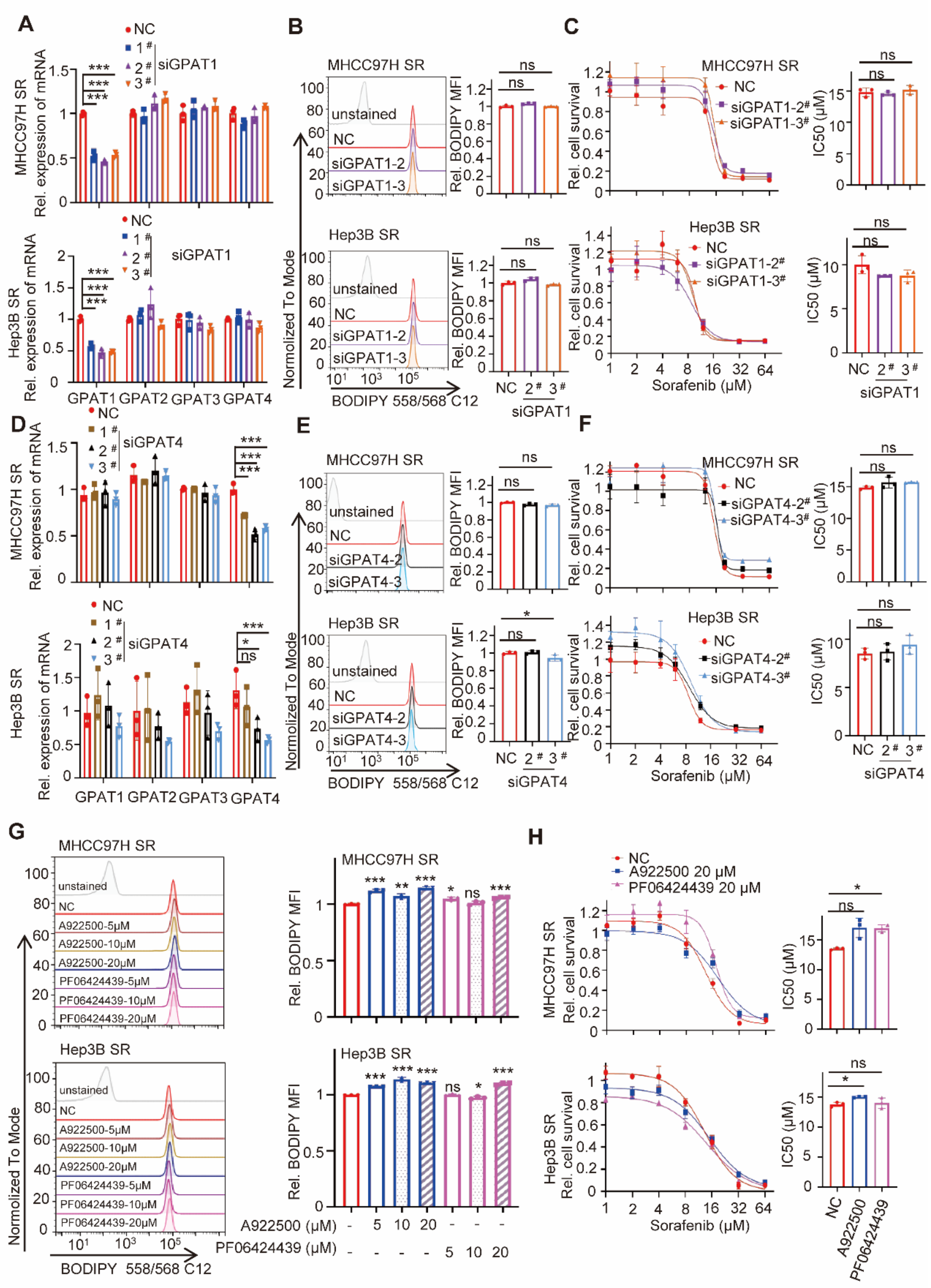

Figure S6. Knocking down DGATs or other GPATs does not affect TAG content and its sensitivity to sorafenib in SR cells. (**A**) mRNA levels of GPATs in NC- and siGPAT1- in MHCC97 and Hep3B SR cells were examined

by RT-qPCR. (**B**) NC- and siGPAT1- in MHCC97 and Hep3B SR cells were stained by Bodipy 558/568 C12, then were determined by flow cytometry. (**C**) The IC_{50} values for sorafenib in MHCC97 and Hep3B SR cells were determined using CCK-8 assay after treatment with various concentrations (1, 2, 4, 8, 16, 32, 64 μM) of sorafenib for 72 hours, with and without siGPAT1. (**D**) mRNA levels of GPATs in NC- and siGPAT4- in MHCC97 and Hep3B SR cells were examined by RT-qPCR. (**E**) NC- and siGPAT4- in MHCC97 and Hep3B SR cells were stained by Bodipy 558/568 C12, then determined by and FCs. (**F**) The IC50 values for sorafenib in MHCC97 and Hep3B SR cells were determined using a CCK-8 assay after treatment with varying concentrations (1, 2, 4, 8, 16, 32, 64 μM) of sorafenib for 72 hours, with and without siGPAT4. (**G**) MHCC97 and Hep3B SR cells treated with different concentrations A922500 or PF06424439 were stained by Bodipy 558/568 C12, then determined by FCs. (**H**) The IC_{50} values for sorafenib in MHCC97 and Hep3B SR cells treated with 20 μM A922500 or PF06424439 were determined using a CCK-8 assay. The cells were exposed to various concentrations (1, 2, 4, 8, 16, 32, 64 μM) of sorafenib for 72 hours. Data are expressed as means ± SEM (n = 3). *$p < 0.05$, **$p < 0.01$, ***$p < 0.001$, ns represents no significant difference.

Figure S7

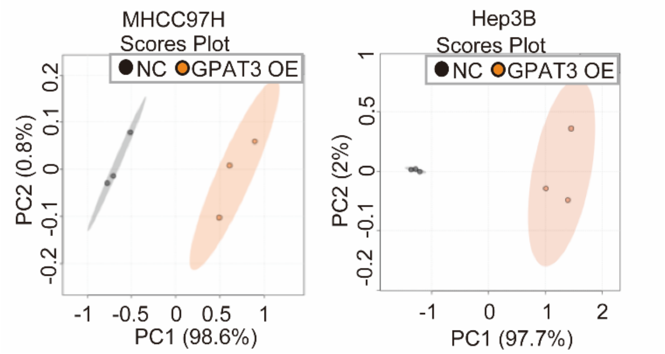

Figure S7. PCA of TAG composition determined by UHPLC-MS/MS in NC- and GPAT3 OE- MHCC97H and Hep3B cells (n=3).

Figure S8

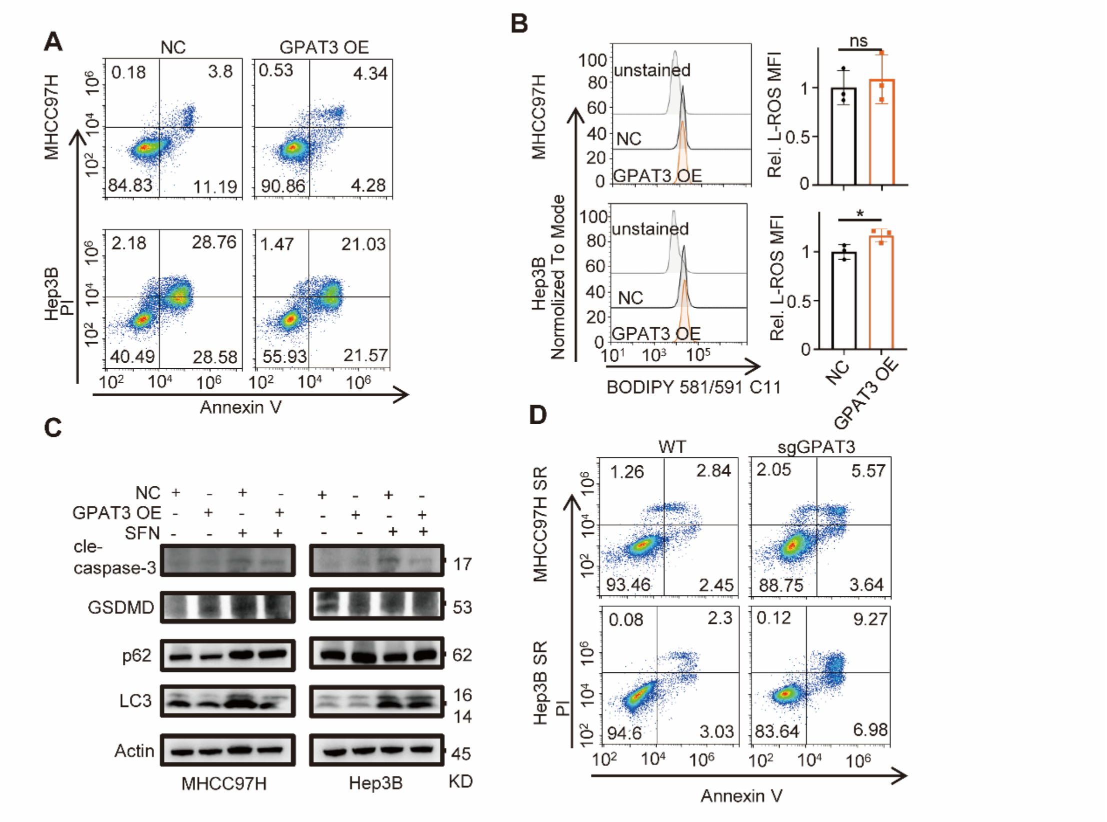

Figure S8. The effect of knockdown or overexpression of GPAT3 to apoptosis. (**A, B**) After treatment with sorafenib (MHCC97H: 10 μM; Hep3B: 6μM) for 72h, cell apoptosis ratios (**A**) and lipid peroxidation levels (**B**) of NC- and GPAT3 OE- MHCC97H or Hep3B cells were determined by FCs. (**C**) After treatment with/without sorafenib (MHCC97H: 10 μM; Hep3B: 6μM) for 72h, protein levels of cle-caspase3, GSDMD, P62 and LC3 in NC- and GPAT3 OE- MHCC97H or Hep3B cells were detected by WB. (**D**) After treatment with sorafenib (MHCC97H: 10 μM; Hep3B: 6μM) for 72h, cell apoptosis ratios of WT- and sgGPAT3- MHCC97H SR or Hep3B SR cells were determined by FCs. Data are expressed as means ± SEM (n = 3). *$p < 0.05$, ns represents no significant difference.

Figure S9

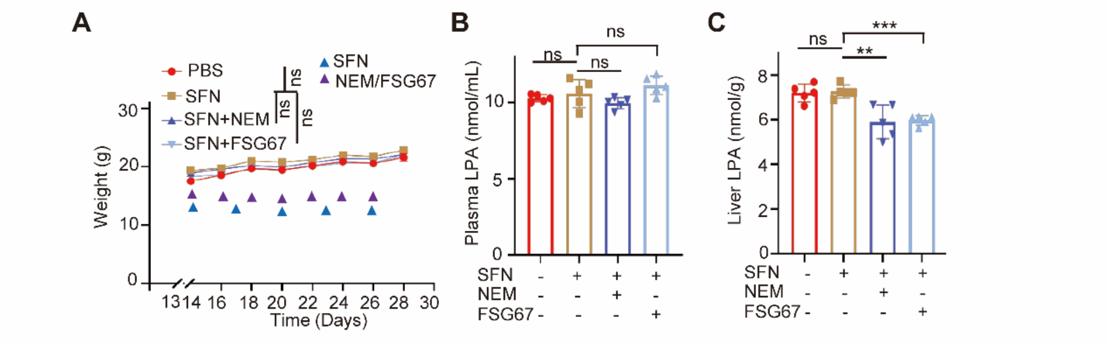

Figure S9. The effects of GPATs inhibitors on mice weight and liver LPA *in vivo*. (**A, B**) Sensitivity of MHCC97H SR cell-transplanted tumors to sorafenib (2 mg/kg, once every 3 days), NEM (1 mg/kg, once every 2 days) and FSG67 (1 mg/kg, once every 2 days) were evaluated *in vivo*. Mice weight (**A**), and ELISA analysis for plasma LPA levels (**B**) and liver LPA levels (**C**). Data are expressed as means ± SEM (n = 5). **$p < 0.01$, ***$p < 0.001$, ns represents no significant difference.
